# Dental injuries in Swiss soccer supporters: A comparative study of regular fans, ultras, and hooligans for public health strategies

**DOI:** 10.1002/cre2.783

**Published:** 2023-09-21

**Authors:** Clarissa Schneider, Michelle Simonek, Florin Eggmann, Andreas Filippi

**Affiliations:** ^1^ Department of Oral Surgery and Dental Traumatology, University Center for Dental Medicine Basel (UZB) University of Basel Basel Switzerland; ^2^ Department of Periodontology, Endodontology and Cariology, University Center for Dental Medicine Basel (UZB) University of Basel Basel Switzerland

**Keywords:** dental trauma, accident prevention, substance intoxication, fan violence, hooligans, mouthguard

## Abstract

**Objectives:**

Violence among soccer supporters continues to pose a significant public health concern in many parts of the world. In Switzerland, hooliganism is largely uninvestigated. This study aimed to examine incidents of violence and associated dental injuries among different groups of soccer supporters, as well as assess the impact of intoxicants on their behavior, using survey data from regular fans, ultras, and hooligans in the Swiss Football League.

**Material and Methods:**

A cross‐sectional survey using a standardized questionnaire was conducted among distinct factions of soccer supporters in the Swiss Football League in 2022. A total of 165 participants self‐identified as belonging to one of three subgroups: “regular fan,” “ultra,” or “hooligan.” Data were gathered on physical altercations, dental injuries, possession of mouthguards, intoxicant use, and medical assistance. Descriptive statistics, logistic regression models, and significance tests were used for data analysis (*α* = .05).

**Results:**

Hooligans had a higher frequency of dental injuries resulting from fights than ultras and regular fans. Hooligans with 11–20 fights per soccer season had a 9.6 times higher probability of dental trauma than those with 0–5 fights (*p* = .048). Possession of a mouthguard was associated with a lower risk of dental injuries for hooligans but an increased risk for ultras. Additionally, hooligans were found to differ significantly from other groups in their consumption of amphetamines and cocaine (*p* < .001).

**Conclusions:**

The study found a strong link between physical altercations and dental injuries among soccer supporters. To promote better prevention, there is a necessity for enhanced educational initiatives facilitated by dentists to amplify the dissemination of mouthguards. Furthermore, it is crucial to raise awareness regarding their proper fitting to minimize the occurrence of combat‐related dental injuries. Health authorities and other stakeholders should take a comprehensive approach to addressing some of the root causes of violent behavior, which include alcohol abuse and illicit substance consumption.

## 1 INTRODUCTION

Football holds a position as the most popular sport in different countries and is strongly emotionally affecting various fan segments (Report from the European Club Association [ECA], 2020). Physical violence has historically been a part of sports, yet in soccer, incidents of off‐field violence are comparatively common. While vandalism and rioting by sports fans have been an issue dating back centuries (Frosdick & Marsh, 2013), these incidents have escalated in frequency since the emergence of soccer culture in Europe during the late 19th century (Dunning et al., 2014; Nepomuceno et al., 2022). The resulting phenomenon known as “football hooliganism” has now attained global prevalence (Brandão et al., 2021; Newson et al., 2018; Spaaij, 2006, 2007) and extended over many European countries (especially Germany and the United Kingdom; Ek & Hooligans, 1996), including Switzerland (Illi, 2004; Sekulic et al., 2015).

In stadia where standing sections exist, “hooligans” and so‐called “ultras” occupy these areas. They define two distinct groups of soccer fans, although some overlap exists. The term “hooligan” dates to the nascent hooligan gangs in the United Kingdom in the 1960s, which were associated with mass violence, public disturbances, and multiple train accidents (Dunning et al., 1982; Nepomuceno et al., 2022). Hooligans represent those with the greatest affinity for violence. They mostly lack any club‐specific identification characteristics; instead, they wear uniform, sporty clothing. Preferred brands in the Swiss scene include Stone Island, Fred Perry, New Balance, and Reeboks (Illi, 2004). The clothing makes it easier for hooligans of opposing teams to find each other outside stadiums and not to involve nonviolent spectators in physical confrontations.

In contrast, the “ultras” are club loyalists and the active core of the stadium spectators using rhythmic clapping, chants, banners, flags, and choreographies. Mostly young club supporters dress in casual‐style dark clothing and deliberately wear only few fan insignia. They are renowned for their unwavering loyalty to their team, often traveling long distances to support them (Doidge & Lieser, 2018).

Before, during, or after soccer matches, physical altercations between opposing groups are not uncommon and remain a pressing issue (Frosdick & Marsh, 2013). There are various theories about fan violence (Reicher and colleagues describe the Elaborated Social Identity Model of Crowd Behavior (Drury & Reicher, 2009; Stott & Reicher, 1998) Newson et al., 2018 the “warrior psychology” as hypotheses). The experience of a profound sense of “oneness” with their fellow fans motivates from time to time also ordinarily “peaceful” supporters to interpret threats to the group as personal threats and become involved in collective conflict (Newson et al., 2018; Stott & Reicher, 1998; Stott et al., 2001). According to Friedmann, 2009, it is difficult to assess the ultras' propensity for violence (Friedmann, 2009). The Swiss police originally categorized the majority of the ultras as peaceful fans; however, physical altercations were increasingly reported. This gave rise to the expression “hooltra” to define the overlap (Brechbühl et al., 2017). Nevertheless, Pilz (2006) refers to violence among ultras as reactive and mentions the concept of territorial defense more than an active seek for confrontation.

The “regular fan” sets themselves apart from these two subgroups by taking on a less active role. They are not affiliated to any groupings and abstain from engaging in violent confrontations, although they regularly attend matches. Sociologist Giulianotti (2002) describes this group as “supporters” as they have a long‐term personal and emotional, sometimes financial, investment in the club. In the stadium, the regular fan often wears the club's colors and partially participates in choirs and chants in a less ardent way.

According to Pilz, those three groups attend matches with distinct personal motivations: regular fans seek commercial entertainment, and ultras follow the game with a critical eye, openly articulating their viewpoints about the club or players (Pilz, 2005; Rasmussen et al., 2012). In contrast, hooligans are primarily propelled by “sensation seeking” (Zuckerman, 1979), embracing conscious experiences of adrenaline kicks, solidarity, courage, and power. Many hooligans characterize these stimulations as being analogous to an addiction (Illi, 2004). That is why the outcome of a match is usually irrelevant to the emergence of hooligan violence. Fights mostly occur as spontaneous clashes between hooligans or violent ultras against opposing fan groups or directed against law enforcement officers before, after, and even beyond the matches. Especially for hooligans, coordinated and premeditated confrontations between opposing hooligans in remote locations far from the stadium are reported. A thesis by the Faculty of the Institute of Sociology in Zurich (Illi, 2004) shows that hooligans follow a strict code of honor: fighting is man‐to‐man, the use of weapons, kicking a fallen attacker or civil prosecution of attackers are frowned upon (Illi, 2004; König, 2002; Wagner, 2002). Injuries during these encounters are often inevitable, but police rarely receive reports (Kett‐Straub, 2012). Sekulic et al. (2015) reported that 86% of 95 Swiss hooligans participating in their survey had experienced dental trauma, and fewer than 40% of them wore dental protection (Sekulic et al., 2015).

Sanctions to prevent violence, including increased police/security presence at games and the implementation of a data collection system (HOOGAN) on violent individuals at sporting events at home and abroad, have been developed over the last years (Press release from the Konferenz der Kantonalen Justiz‐ und Polizeidirektorinnen und ‐direktoren, 2016), which reinforced the notorious secretiveness of the scene toward outsiders, the media or the police. The Swiss hooligan scene is largely uninvestigated and considered relatively small (approximately 250 individuals [2004]; Illi, 2004), with the active scenes primarily located in the cities of Basel, Zurich, Bern, and Lugano.

The objective of this study is to examine the occurrence of violent incidents among soccer supporters, measured by the frequency of physical altercations per season, focusing on associated dental injuries, and possession of protective mouthguards. The authors hypothesized differences between these three groups in the frequency and severity of dental injuries, substance abuse, and possession of protective equipment. Additionally, the study aimed to assess the impact of intoxicants on soccer supporter behavior. As alcohol and certain drugs are known to increase the willingness for violence (Denison et al., 1997; Kuypers et al., 2020), higher consumption was expected among the more violent fans. Moreover, data regarding spectators' perceptions of post‐COVID‐19 changes in the stadium were collected.

The investigation draws upon survey data from three distinct cohorts of stadium attendees in the Swiss Football League: hooligans, ultras, and regular fans, and compares their responses.

## 2 MATERIALS AND METHODS

A cross‐sectional retrospective study, duly authorized by the Swiss Association of Research Ethics Committees (Swissethics), was conducted from August to October 2022 to investigate violence among distinct factions of soccer supporters within the Swiss Football League.

A total of 165 participants from different Swiss football teams were personally interviewed at and around soccer matches using a standardized questionnaire (in German) after being assured of complete anonymity. The interviewees self‐identified as belonging to one of three subgroups after giving them three options to choose from: “regular fan,” “ultra,” or “hooligan.” An advantage in achieving access to the hooligan and ultra groups was gained through direct contact and good connections to the scene through private networks. Individuals who did report to not regularly attend Swiss Football League matches were excluded from the study.

The standardized multiple‐choice questionnaire consisted of questions on age, frequency of physical altercations per season, resulting dental injuries, mouthguards, intoxicant use, utilization of medical assistance, and spectators' perceptions of post‐COVID‐19 changes (Table 1). If a mouthguard was available, a photo was requested on‐site. The investigators classified the mouthguards according to the following categories: custom‐made mouthguards, self‐individualized mouthguards, and not individualized mouthguards. To facilitate the terms for the layman, the dental injuries were classified as “broken,” “loose,” and “loss.”

**Table 1:** Questionnaire.

Questionnaire item	
1. Age group	<20/21–30/31–40/≥40
2. Fan group affiliation^a^	Regular fan/ultra/hooligan
3. Number of fight involvements before, during, or after soccer games per season^a^	0/1–5/6–10/11–20
4. Dental injury	Yes/no
5. Type of dental injury^b^	Tooth loss (avulsion)/loose or displaced tooth (luxation)/tooth fractures (crown fracture, crown‐root fracture)
6. Number of times a dental injury was sustained^a^	1/2/>2
7. Dental visit because of the dental injury^b^	Immediately/on the same day/delayed/none
8. Mouthguard	Yes/no
9. Why do you own a mouthguard?^a^	Already had one for recreational sport activities/upon recommendation by others/after previous injury/own initiative
10. Type of mouthguard?^a^	Custom‐made/self‐individualized/not‐individualized
11. Soccer game‐related alcohol and/or drug use^b^	Alcohol/cocaine/cannabis/amphetamines/others
12. Cocaine use^b^	At soccer games/for fights/habitual use on a regular basis
13. Did the illegal background of an injury ever hold you back from seeking medical/dental help?^a^	Yes/no
14. Did you notice any change after the post‐pandemic reopening?^a^	Yes/no
15. What did you percieve?^b^	More visitors/less visitors/more fights/less fights

^a^ Single‐choice question.

^b^ Multiple‐choice question.

Descriptive statistics were used to describe categorical variables, indicating the frequency and proportion in each group. Two‐sided significance tests such as Fisher's exact tests or *Χ* ^2^ tests were performed, and logistic regression models were used to predict dental injuries (or type of injury) between the groups, with “no injury” as the reference. The resulting estimates were presented as odds ratios (OR) with the corresponding 95% confidence interval (CI) and *p*‐values. A *p* < .05 was considered significant. All analyses were performed by an unblinded statistician using R software (version 4.2.1) (R Core Team; R Foundation for Statistical Computing).

## 3 RESULTS

The study participants comprised 165 individuals, comprising 38 hooligans, 60 ultras, and 67 regular fans, mainly between the ages of 21 and 40 years (66 interviewees 21–30 years old, 65 interviewees 31–40 years old).

When asked about their frequency of involvement in fights per soccer season, it was found that six hooligans reported being involved in 11–20 fights, while the remaining 32 hooligans reported involvement in 1–10 fights. Two‐thirds of ultras did not report any involvement in fights, and fights among regular fans were nearly nonexistent. Within the cohort studied, it was found that a minority of ultras (eight out of 60) suffered fight‐related dental injuries, while nearly half of the hooligans had already experienced at least one (*n* = 11) or even multiple (*n* = 7) dental injuries. Tooth fractures, including crown fractures and crown‐root fractures, and luxations were the most common injuries across all groups, while avulsions were exclusively reported by hooligans.

Logistic regression models revealed that hooligans with 11–20 fights had a 9.6 times higher probability of suffering dental trauma than those with 0–5 fights (*p* = .048) (Table 2). Ultras with 1–10 fights had a 22‐fold higher probability of suffering dental trauma than ultras who did not participate in fights (*p* = .005) (Table 2). It was found that above a quarter (10/38) of hooligans forwent medical help due to illegal background of the injury, which is more likely than ultras or regular fans. Immediate and delayed visits to dentists were reported by respondents across all groups.

**Table 2:** Odds ratio and 95% confidence interval.

	OR (95% CI)
Fights ultras	
1–10 compared to 0	22.2 (2.58, 190.83)
Fights hooligans	
11–20 compared to 0–5	9.6 (1.02, 90.34)
6–10 compared to 0–5	1.2 (0.31, 4.59)
Dental injury ultras	
Mouthguard compared to no mouthguard	7 (1.17, 41.74)
Dental injury hooligans	
Mouthguard compared to no mouthguard	0.64 (0.18, 2.24)
Risk of loose tooth	
Mouthguard compared to no mouthguard	16.33 (0.81, 330.35)
Risk of chipped tooth	
Mouthguard compared to no mouthguard	5.44 (0.75, 39.43)

Two‐thirds of the hooligans (25/38) and some ultras (5/60) and regular fans (11/67) were in possession of a mouthguard. It was observed that many participants possessed mouthguards not only for soccer‐associated events but due to their participation in other recreational sports activities (“already had one”) or upon recommendation by others (16/41). Mouthguards were obtained after a previous dental trauma by three participants, and six others obtained them on their own initiative. Hooligans in possession of a mouthguard had a 1.6 times lower likelihood (OR: 0.64) of sustaining dental injuries than hooligans without one (*p* = .49) (Table 2). Furthermore, they were less likely to suffer tooth loss, luxations, or tooth fractures (*p*: .87, .12, and .96). Ultras with mouthguards had up to a sevenfold greater risk of tooth injury compared with those without mouthguards (*p* = .033) (Table 2).

Photographs of 18 mouthguards were obtained exclusively from ultras and hooligans. Most mouthguards were self‐individualized, while only three were custom‐made (Table 3).

**Table 3:** Results of the questionnaire‐based survey.

	Regular fan^a^	Ultra^a^	Hooligan^a^	p value
**Number of study participants**	67	60	38	
**Fights per soccer season**				<.001
0	61 (91)	38 (63.3)	1 (2.6)	
1–5	6 (9)	21 (35)	19 (50)	
6–10	0 (0)	1 (1.7)	12 (31.6)	
11–20	0 (0)	0 (0)	6 (15.8)	
**Type of dental injury**				<.001
None	66 (89.5)	52 (83.9)	20 (44.4)	
Tooth fractures	1 (1.5)	8 (12.9)	13 (28.9)	
Luxation	0 (0)	2 (3.2)	6 (13.3)	
Avulsion	0 (0)	0 (0)	6 (13.3)	
**Visit to the dentist after a dental injury**				
Immediately	1 (1.5)	0 (0)	2 (5.3)	
On the same day	0 (0)	1 (1.7)	2 (5.3)	
Delayed	0 (0)	4 (6.7)	9 (23.7)	
None	0 (0)	3 (5)	4 (10.5)	
**Mouthguard**				<.001
None	56 (83.6)	55 (91.7)	13 (34.2)	
Not‐individualized	0 (0)	1 (1.7)	0 (0)	
Self‐individualized	10 (14.9)	4 (6.7)	23 (60.5)	
Custom‐made	1 (1.5)	0 (0)	2 (5.3)	

^a^ Percentage displayed in parentheses.

**Figure 1:**
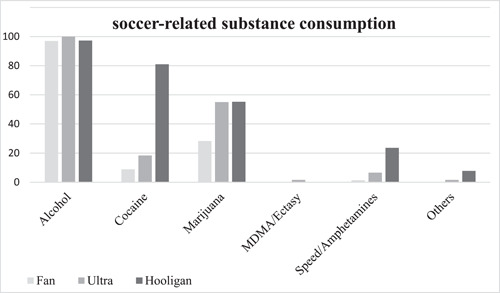
Consumption of various drugs in the context of soccer matches among fans, ultras, and hooligans.

Hooligans were found to differ significantly from the other groups (*p* < .001) in their consumption of amphetamines (9/38) and cocaine (30/38). Hardly any ultras and regular fans reported using these substances (Figure 1). Cocaine was used by most hooligans (30/38) also in a habitual manner (22/30). In the context of soccer matches, the motivation for cocaine consumption was equally divided between consumption during fights (8/38) and consumption related to the game (7/38).

Almost one‐third of the spectators (62/165) perceived their stadium experience unchanged post‐COVID‐19. Seventy‐four others noticed fewer spectators, while 29 noticed more. Responses on physical violence varied: 37 perceived fewer fights, but 22 reported more, revealing controversy.

## 4 DISCUSSION

This study investigated patterns of violence among soccer supporters, specifically fight‐related dental injuries in the context of soccer matches among regular fans, ultras, and hooligans. Additionally, the study examined the potential protective effects of dental equipment and the general consumption of different intoxicants.

Hooligan violence remains an ongoing security matter that the Swiss federal government still pays attention to (Bundesamt für Polizei fedpol, 2023). Hooligans were found to engage in regular fights more frequently than ultras, with sustained fights numbering between 6 and 10 or between 11 and 20 per season. Conversely, ultras generally fought less frequently, with only 21 out of 60 surveyed ultras engaging in 1–5 fights per season. Comparing the survey data with findings from a cross‐sectional study conducted in 2012 and 2013 (Sekulic et al., 2015), the study found no significant changes in the propensity for violence over the past decade, with the participation rate in regular fights remaining consistent between the 2012/13 and 2022 seasons. Notably, 6/38 of participants engaged in 11–20 fights in both seasons. While slightly less frequent than a decade ago, two‐thirds of hooligans still participated in 6–10 fights during the 2022 season (Sekulic et al., 2015).

The Swiss Federal Office of Police (fedpol) has been collecting statistics on violent incidents during football matches, both domestically and internationally, for several years. Over the last 5 years, a relatively consistent number of violent events have been recorded (Bundesamt für Polizei fedpol, 2023). These findings align with the results of the survey conducted in this study: 62/165 did not notice any changes, and similar proportions of decreased (37/165) and increased (22/165) rates of fights have been reported.

The risk of dental injury increased with the frequency of fight involvement. This study's findings revealed a 10‐fold increase in the probability of dental injury in hooligans and a remarkable 20‐fold increase in ultras compared with non‐fighting or low‐fighting study participants. It is noteworthy that avulsions were exclusively observed in hooligans despite a lower overall risk of injury compared with ultras. The increased frequency, as well as the severity of the altercations could explain this result. The most frequently observed injury patterns were tooth fractures and luxations, which is consistent with the results of a previous study conducted 10 years ago by Sekulic et al. (2015) where “displacements” and “broken/chipped” were also described as the most common injuries.

The use of mouthguards has become increasingly prevalent in various sports, and they are mandatory or commonly used in combat sports such as boxing, kickboxing, and mixed martial arts (Holmes & Holmes, 2000; Ifkovits et al., 2015; Mańka‐Malara et al., 2022; Shimoyama et al., 2009; Tulunoglu & Ozbek, 2006; Zürcher et al., 2020). It is worth noting that many hooligans participate in recreational martial arts activities to the extent of organized competitions (Sekulic et al., 2015). However, in a survey conducted a decade ago, less than 40% of hooligans reported owning a mouthguard (Sekulic et al., 2015). In the present study, a significant increase in the adoption of mouthguards among hooligans was observed, with 66% of participants reporting possession. This encouraging trend could potentially reduce the risk of dental injuries associated with physical altercations among this population. In addition to the general need for violence prevention, the results underscore the importance of promoting the use of protective gear, including mouthguards, among violent hooligans and ultras. Hooligans who reported possessing mouthguards had a significantly lower risk of sustaining dental injuries during fights. Interestingly, the analysis showed that ultras who reported using mouthguards had a higher risk of injury. One possible explanation for this observation is that ultras who own mouthguards may be more willing to engage in physical altercations than nonfighting ultras who actively avoid confrontations. Additionally, as ultras generally participate in fewer fights than hooligans, they may have less experience in such altercations, leading to a higher risk of injury even with dental protection. Further research is needed to evaluate whether even a sufficient mouthguard can prevent injuries in individuals with limited experience in fighting.

Some of the fans possessed mouthguards, commonly used for recreational sports, and might have been involved in minor brawls. The presence of regular fans in some fights possibly indicates an overlap between ultras and regular fan groups. It is also possible that ultras tried to maintain anonymity and conceal their true identity by self‐identifying as a regular fan.

**Figure 2:**
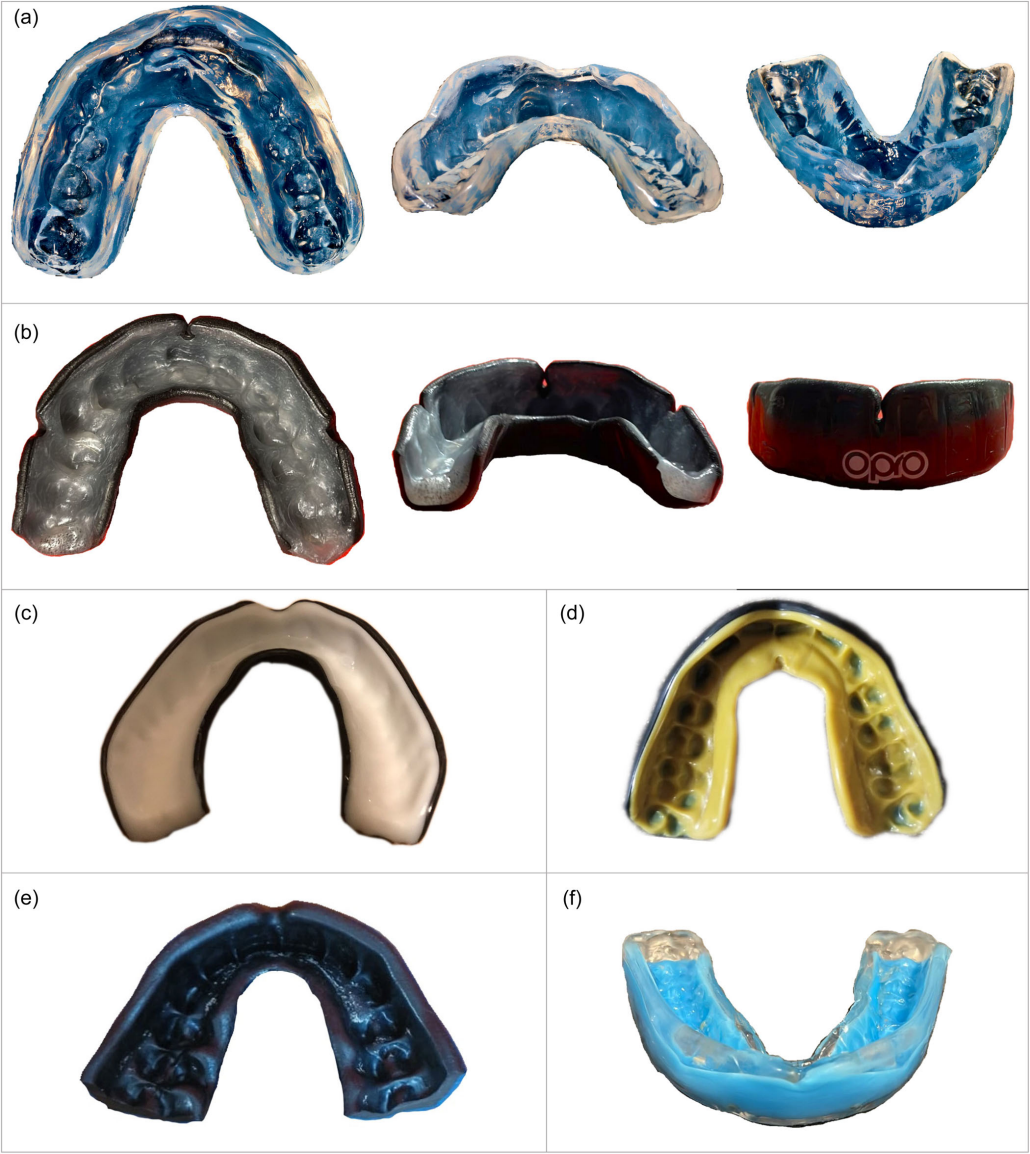
(a–f) Examples of mouthguards photographed on‐site, along with their respective characteristics (a, custom‐made mouthguard; b, self‐individualized mouthguard; c, not‐individualized mouthguard; d, reduced occlusal thickness; e, reduced distal length; f, insufficient height of the labial flange).

Mouthguards protect against serious oral injuries by distributing and absorbing the force through the flexible polymeric material. Custom‐made mouthguards (Figure 2a) are adapted in a dental laboratory to a cast from an impression by a dentist. The optimal guard design should enclose the maxillary teeth, including the first molars (Figure 2e shows a negative example). The optimal thickness of a mouthguard should be determined based on the sport and expected level of impact force, but generally falls within the range of 4–8 mm. Additionally, the labial flange should extend up to the vestibule (Figure 2f illustrates a negative example), while the palatal flange should extend approximately 4 mm. It is also recommended that maxillary guards be articulated against the mandibular model to ensure proper fit and protection (Lang & Filippi, 2003; Zürcher et al., 2020). Self‐individualized mouthguards (Figure 2b) are typically adjusted using the “boil‐and‐bite” technique, which involves heating the mouthguard and molding it by biting and sucking. However, if the mouthguard is bitten too hard during the fitting process, it may result in a poorer fit and thinner material over prominent teeth and occlusal surfaces, ultimately leading to reduced protection (as shown in Figure 2d). Conversely, not‐individualized mouthguards (Figure 2c) come in various sizes and are ready to use, but they provide the poorest fit and minimal protection. In fact, they may be considered dangerous as they can give the user a false sense of security (Patrick et al., 2005).

Alcohol and other intoxicants have significant effects on the psychomotor system, altering cognitive abilities and perception. Fear and threat awareness are dampened, the sensation of pain decreases (Hoaken & Stewart, 2003), the responsiveness to nonverbal cues reduces (Attwood & Munafò, 2014; Melkonian & Ham, 2016), and the willingness to engage in risky behavior increases (Miller & Fillmore, 2014). Several studies have demonstrated that blood alcohol levels above a certain concentration can facilitate aggression (Chermack & Giancola, 1997; De Sousa Fernandes Perna et al., 2016; Duke et al., 2011; Giancola, 2002; Kuypers et al., 2020). On the other hand, cannabis is known primarily for its calming effect and enhancement of prosocial behavior (De Sousa Fernandes Perna et al., 2016; Silva et al., 2022; Vigil et al., 2022). Mixed consumption of alcohol and drugs, however, can potentially exacerbate the negative effects (Kuypers et al., 2020).

The available evidence on the effects of cocaine use on violent human behavior is limited. In a study involving cocaine‐dependent veterans, the use of cocaine did not appear to elicit behavior that had not been previously exhibited (Denison et al., 1997). However, the likelihood of cocaine users engaging in violent behavior can increase with the additional use of alcohol (Denison et al., 1997), and this risk is higher with higher cocaine dosages (Licata et al., 1993). Although there is no direct evidence linking amphetamine use and aggression, studies suggest that amphetamines generally cause lightheadedness and elevated energy levels while reducing natural fear‐intensified reactions in response to unpleasant stimuli (Chitty et al., 2014; Corr & Kumari, 2013). Combined with alcohol, amphetamine use can increase the risk of unprovoked violent behavior (Harro, 2015; Kuypers et al., 2020). In general, the use of alcohol and intoxicants, particularly in combination, increases the risk of becoming a victim or perpetrator of violence. The present study revealed that fans and ultras were more likely to use cannabis in conjunction with alcohol, whereas more violent hooligans were more likely to use stimulant substances such as cocaine and amphetamines.

Moreover, the unlawful background of an injury, such as drug use, was the reason for more than a quarter (10/38) of the hooligans' reluctance to seek medical assistance, including dental care. Preventative measures such as education and outreach programs are therefore crucial to address substance abuse and reduce the risk of violent behavior among soccer supporters.

The present study has several limitations that should be considered when interpreting the results. First, information bias may have been introduced due to the self‐report nature of the data collection and the respondents' self‐assignment into one of the three groups. To avoid response biases in an interview‐based study design, we ensured complete anonymity. The interviews were conducted individually and without the presence of third parties. As some interviews were facilitated through personal contacts, we anticipate that responses were provided as honest as possible. Furthermore, the data collected did not include information on whether the participants were wearing a mouthguard at the time of dental trauma, which may have affected the severity of the injuries sustained. Another limitation is that the number of participants in the “hooligan group” was notably smaller than the other two groups due to the clandestine nature of this population and fear of prosecution, leading to difficulties in recruiting participants.

## 5 CONCLUSION

The findings of this study indicate a strong link between the frequency of physical altercations and the prevalence of dental injuries. The results underscore the importance of promoting the use of protective gear, including mouthguards, among violent hooligans and ultras to reduce the frequency and severity of injuries. It is incumbent upon the football clubs, police, and transport companies to continue to implement and update their policies in line with the 2007 Concordat on Measures to Prevent Violence at Sporting Events (Press release from the Konferenz der Kantonalen Justiz‐ und Polizeidirektorinnen und ‐direktoren, 2016). As the propensity for violence has not changed significantly over the past 10 years, health authorities and other stakeholders should comprehensively address the underlying factors driving violent conduct, including alcohol abuse and illicit substance use.

## AUTHOR CONTRIBUTIONS

Clarissa Schneider and Michelle Simonek contributed equally to the interviews, conceptualization, and writing. Andreas Filippi contributed to project administration, supervision, and reviewing. Florin Eggman is involved in Language editing and script translation.

## CONFLICT OF INTEREST STATEMENT

The authors declare no conflict of interest.

## ETHICS STATEMENT

The study was granted approval by the Swiss Association of Research Ethics Committees (Swissethics) and assigned the BASEC reference number Req‐2022‐01226.

## Data Availability

The data that support the findings of this study are available from the corresponding author upon reasonable request.
